# Mutations in the nuclear localization signal of nsP2 influencing RNA synthesis, protein expression and cytotoxicity of Semliki Forest virus

**DOI:** 10.1099/vir.0.83320-0

**Published:** 2008-03

**Authors:** Kristi Tamm, Andres Merits, Inga Sarand

**Affiliations:** 1 Estonian Biocentre, Riia 23, 51010, Tartu, Estonia; 2 Institute of Technology, University of Tartu, Nooruse 1, 50411, Tartu, Estonia

## Abstract

The cytotoxicity of Semliki Forest virus (SFV) infection is caused partly by the non-structural protein nsP2, an essential component of the SFV replicase complex. Due to the presence of a nuclear localization signal (NLS), nsP2 also localizes in the nucleus of infected cells. The present study analysed recombinant SFV replicons and genomes with various deletions or substitutions in the NLS, or with a proline-to-glycine mutation at position 718 of nsP2 (P718G). Deletion of one or two arginine residues from the NLS or substitution of two of the arginines with aspartic acid resulted in a virus with a temperature-sensitive phenotype, and substitution of all three arginines was lethal. Thus, most of the introduced mutations severely affected nsP2 functioning in viral replication; in addition, they inhibited the ability of SFV to induce translational shut-off and kill infected cells. SFV replicons with a P718G mutation or replacement of the NLS residues ^648^RRR^650^ with RDD were found to be the least cytotoxic. Corresponding replicons expressed non-structural proteins at normal levels, but had severely reduced genomic RNA synthesis and were virtually unable to replicate and transcribe co-electroporated helper RNA. The non-cytotoxic phenotype was maintained in SFV full-length genomes harbouring the corresponding mutations; however, during a single cycle of cell culture, these were converted to a cytotoxic phenotype, probably due to the accumulation of compensatory mutations.

## INTRODUCTION

Semliki Forest virus (SFV; genus *Alphavirus*) is an enveloped, positive-strand RNA virus. Its genome of approximately 11.5 kb with a 5′ cap and 3′ poly(A) tail encodes four non-structural proteins (nsP1–4) involved in viral RNA synthesis and five structural proteins. After virus entry into the cell, the non-structural region is translated directly from genomic RNA into a large non-structural polyprotein, which is processed into an early and subsequently a late replicase complex (Strauss & Strauss, 1994). The early replicase complex mediates synthesis of a negative-strand RNA complementary to the genomic RNA. Subsequently, minus strands are used by the late replicase complex as templates for new genomic and subgenomic RNAs (sgRNAs). The latter are used for the translation of structural proteins. In the replicon vector system, the coding region of structural genes is replaced with a polylinker and/or foreign gene sequences. The replicon RNA is self-replicating and can be packaged into virus-like particles (VLPs) if the genes encoding the structural proteins are provided by additional helper RNA (Liljestrom & Garoff, 1991).

nsP2 is an essential and multifunctional component of the viral replicase complex. Its N-terminal domain has NTPase, RNA triphosphatase and RNA helicase activities (Gomez de Cedron *et al.*, 1999; Rikkonen *et al.*, 1994a; Vasiljeva *et al.*, 2000). The C-terminal domain of nsP2 is responsible for the processing of non-structural polyprotein (Strauss & Strauss, 1994). nsP2 has also been reported to be essential for the cessation of negative-strand RNA synthesis in the late stage of the infection cycle and for the regulation of sgRNA synthesis, possibly through stabilization of the late replicase complex. Mutations in nsP2 result in unstable late replicase complexes, a severe reduction in viral RNA synthesis and in continuous minus-strand synthesis in infected BHK-21 cells (Sawicki *et al.*, 2006).

In susceptible vertebrate cells, alphavirus infection strongly affects cellular metabolism, causing inhibition of transcription and translation, and cell death. Alphavirus-induced shutdown of host-cell transcription and translation involves separate processes (Gorchakov *et al.*, 2005), and nsP2 plays a major role in these processes for Old World alphavirus infections (Garmashova *et al.*, 2007). Mutations in the C terminus of nsP2 of SFV or Sindbis virus (SIN) are often responsible for persistent infection or prolonged survival of infected cells (Dryga *et al.*, 1997; Frolov *et al.*, 1999; Perri *et al.*, 2000), and have been used to construct alphavirus-based replicons with reduced cytotoxicity (Agapov *et al.*, 1998; Lundstrom *et al.*, 2001, 2003).

In SFV-infected cells, approximately half of the nsP2 is transported to the nucleus (Peranen *et al.*, 1990; Rikkonen, 1996). The nuclear localization of nsP2 has been proposed as a trigger for transcriptional shutdown and apoptosis of infected cells (Rikkonen *et al.*, 1994b). The pentapeptide ^648^PRRRV^652^ has been identified as the nuclear localization signal (NLS) of nsP2. Replacement of arginine 649 with aspartic acid changed the localization of nsP2 to entirely cytoplasmic (Rikkonen *et al.*, 1992). SFV harbouring the corresponding mutation was viable (Rikkonen *et al.*, 1994b) and showed attenuated neuropathogenicity, resulting in slower viral spread and reduced cell death in infected adult mice brain neurons (Fazakerley *et al.*, 2002). However, this alteration in NLS did not prevent apoptosis in infected BHK-21 cells.

In the present study, we compared different NLS mutants of nsP2 to determine the residues critical for SFV RNA synthesis, gene expression and virus-induced cytotoxicity, and investigated possible underlying mechanisms. The non-cytotoxic phenotype correlated with reduced accumulation of SFV RNA. All SFV mutants that retained the cytotoxic phenotype also retained some transport of nsP2 into the nucleus, but not necessarily vice versa. Thus, it is hypothesized that at least two events – nuclear localization of nsP2 and a sufficiently high accumulation of viral RNA – are necessary for cytotoxicity.

## METHODS

### Cells and medium.

BHK-21 cells were grown in Glasgow minimal essential medium containing 5 % fetal calf serum, 0.3 % tryptose phosphate broth, 0.1 U penicillin ml^−1^ and 0.1 μg streptomycin ml^−1^. All cells were grown in an incubator at 28 °C with 5 % CO_2_.

### Construction of SFV mutant replicons, genomes and helper constructs.

All recombinant viruses, replicons and helper RNAs were based on the previously described plasmids pSP6-SFV4 (Liljestrom *et al.*, 1991), pSFV1, pHelper1 (Liljestrom & Garoff, 1991) and pHelperC (Smerdou & Liljestrom, 1999). Mutations designated DDD, RDD, DDR, RDR, RRD, ΔΔΔ, ΔΔR and ΔRR were introduced into the NLS of nsP2 (^648^RRR^650^) by PCR-based mutagenesis. Mutated DNA fragments were introduced by subcloning into pSFV1-d1EGFP (Zusinaite *et al.*, 2007) and the resulting replicon plasmids were designated pSFV1-d1EGFP-DDD, pSFV1-d1EGFP-RDD, pSFV1-d1EGFP-DDR, pSFV1-d1EGFP-RDR, pSFV1-d1EGFP-RRD, pSFV1-d1EGFP-ΔΔΔ, pSFV1-d1EGFP-ΔΔR and pSFV1-d1EGFP-ΔRR. The replacement of proline for glycine at the position 718 of nsP2 was performed similarly and the corresponding replicon plasmid was designated pSFV1-d1EGFP-PG. For some assays, the polylinker–d1EGFP region of these plasmids was replaced by an expanded multiple cloning site (MCS), by the firefly luciferase sequence (Luc), by the SFV capsid enhancer and the foot-and-mouth disease virus 2A autoprotease and the luciferase sequence (EnhLuc) or by the puromycin *N*-acetyltransferase sequence (Pac); corresponding replicon plasmids were designated pSFV1-MCS, pSFV1-Luc, pSFV1-EnhLuc and pSFV1-Pac, respectively.

Plasmids containing full-length infectious cDNA of SFV4-RDD, SFV4-DDR, SFV4-RDR, SFV4-RRD, SFV4-ΔΔR, SFV4-ΔRR and SFV4-PG were created by replacement of the *Bgl*II–*Spe*I fragment in the corresponding replicon plasmids originating from pSFV1-d1EGFP by the *Bgl*II–*Spe*I fragment from pHelper1.

To construct helper RNAs expressing marker proteins, the coding sequence of the structural proteins from pHelper1 was replaced by a polylinker. The sequences encoding Luc or EnhLuc were transferred to this polylinker from pSFV1-Luc and pSFV1-EnhLuc, respectively. The resulting plasmids were designated pH-SG-Luc and pH-SG-EnhLuc. Sequences of all primers and constructed plasmids are available upon request.

### RNA transcription and transfection.

SFV-based replicon plasmids, helper plasmids and infectious cDNA plasmids were linearized by *Spe*I digestion. The RNAs were synthesized *in vitro* using SP6 RNA polymerase and used for cell transfection by electroporation as described by Karlsson & Liljestrom (2003); transfection efficiency was >95 % in all experiments. For VLP production, BHK-21 cells were co-transfected with equal amounts of *in vitro*-transcribed Helper1 RNA and replicon RNAs.

### Collection of virus stocks and VLPs.

Primary virus stocks or VLPs were collected from transfected BHK-21 cell cultures after incubation at 28 °C for 72 h. Virus titres were determined by plaque assay. VLP titres were determined by infecting BHK-21 cells with different dilutions of VLP stocks in serum-free media and counting d1EGFP-positive cells at 48 h post-infection (p.i.).

### Immunofluorescence microscopy.

BHK-21 cells were grown on cover slips and infected with virions in serum-free medium at an m.o.i. of 1; mock-infected cells were used as controls. At 8 or 24 h p.i., cells were washed with PBS, fixed with 4 % paraformaldehyde for 10 min at room temperature and permeabilized with cold methanol for 6 min at –20 °C. Cells were washed with PBS, blocked in 5 % FCS in PBS and incubated for 1 h with rabbit polyclonal antibody against SFV nsP2. Alexa Fluor 488 (Invitrogen)-labelled anti-rabbit antibody was used as a secondary antibody. Samples were analysed on a Nikon D-Eclipse C1 confocal microscope.

### Analysis of cytotoxicity of SFV vectors.

The cytotoxicity of SFV vectors was analysed essentially as described by Garmashova *et al.* (2006). Five micrograms of *in vitro*-synthesized RNA from pSFV1-Pac-type vectors was electroporated into 10^6^ BHK-21 cells; mock-transfected cells were used as controls. The cells were seeded into 24-well culture plates (growth area 2.0 cm^2^ per well) and puromycin selection (10 μg ml^−1^) was applied from 16 h post-transfection (p.t.). Numbers of viable adherent cells were determined at 2, 24, 48, 93, 141, 237 and 312 h p.t. using trypan blue (Flow Laboratories) exclusion.

### Metabolic labelling.

BHK-21 cells were transfected with 50 μg *in vitro*-synthesized RNA or infected with virus particles at an m.o.i. of 1. For each transfection, 5×10^6^ cells were used. Depending on the experiment, transfected cells were seeded into four or six 35 mm diameter plates. For infection, 2×10^5^ cells on 35 mm plates were used for each time point. At selected times, transfected or infected cells were washed twice with PBS and once with methionine- and cysteine-free Dulbecco's modified Eagle's medium, followed by incubation for 1 h in the latter medium. The cells were then labelled with 50 μCi [^35^S]methionine and [^35^S]cysteine (RedivuePRO-MIX; Amersham Biosciences) ml^−1^ for 1 h. After labelling, the cells were washed with PBS, lysed in 50 μl Laemmli buffer and subjected to 12 % SDS-PAGE; 5 μl lysate was loaded into each well.

### Pulse–chase experiments and immunoprecipitation.

Polyprotein processing was studied by pulse-labelling of the cells with [^35^S]methionine. BHK-21 cells (10^6^) were infected at an m.o.i of 5 and labelled at 6 h p.i. for 0.5 h. In chase samples, the pulse was followed by a chase for 1 h in the presence of excess of unlabelled methionine. Cells were then lysed in 1 % SDS and the proteins denatured by boiling. Samples were diluted 1 : 20 with NET buffer [50 mM Tris/HCl (pH 7.5), 150 mM NaCl, 5 mM EDTA, 0.5 % NP-40] and incubated for 1 h at 4 °C with rabbit polyclonal antiserum against non-structural proteins (Salonen *et al.*, 2003). Immunocomplexes were precipitated with Protein A–Sepharose CL-4B (Amersham Biosciences) overnight at 4 °C. The precipitates were washed four times with NET buffer containing 400 mM NaCl. The precipitated proteins were denatured by heating in Laemmli buffer and the samples were separated by SDS-PAGE. The labelled proteins were detected by autoradiography.

### Quantification of luciferase expression and Western blotting.

For *in cis* expression assays, 2×10^5^ BHK-21 cells were transfected with 2 μg SFV1-Luc-type or SFV1-EnhLuc-type RNA; for *in trans* expression assays, combinations of SFV1-MCS-type RNA with H-SG-Luc or H-SG-EnhLuc RNA (2 μg each) were used. Electroporated cells were seeded into two wells (growth area 2.0 cm^2^ per well) and incubated for 24 h at 28 °C. Cells from one well were lysed and analysed using the Luciferase Assay System (Promega) on a Turner Designs luminometer. Cells from another well were lysed using Laemmli buffer and the expressed proteins were subjected to 12 % SDS-PAGE. Proteins were transferred to a nitrocellulose membrane and probed either with rabbit polyclonal antisera against SFV nsP1 or with a mouse monoclonal antibody against *β*-actin (Abcam). Western blots were visualized using an ECL Immunoblot Detection kit (Amersham Life Science).

### Northern blot analysis.

BHK-21 cells (5×10^6^) were co-transfected with 6 μg SFV1-MCS-type RNA and 6 μg H-SG-EnhLuc RNA. Transfected cells were divided among three plates and incubated at 28 °C for 24, 48 or 72 h. Total RNA from transfected cells was purified with TRIzol reagent (Invitrogen). Equal amounts of total RNA were denatured for 10 min at 65 °C in formamide/formaldehyde buffer and separated by electrophoresis in 1.5 % agarose gel supplemented with 0.2 M formaldehyde. The separated RNAs were transferred to Hybond-N+ membrane (Amersham Biosciences) and UV cross-linked. Hybridization was performed using a standard procedure. The bound radioactivity was quantified using Typhoon TRIO equipment and ImageQuant TL software (Amersham Biosciences).

## RESULTS

### Phenotypes of mutant replicons

Information on the role of the nsP2 NLS in SFV-induced cytotoxicity is limited (Rikkonen, 1996; Rikkonen *et al.*, 1992). In order to screen the NLS sequence of nsP2 for changes with potential impact on SFV cytotoxicity, we constructed and analysed six novel mutant replicons and viruses carrying substitutions/deletions in the ^648^RRR^650^ sequence of nsP2. These included substitution of two or three arginines with aspartic acids (DDD, RDD and DDR), or complete or partial deletion of the ^648^RRR^650^ sequence (ΔΔΔ, ΔΔR and ΔRR). Two previously described mutants (RDR and RRD; Rikkonen, 1996; Rikkonen *et al.*, 1992) were also included in the study. In addition, an nsP2 mutant with a ^718^P→G substitution was constructed. The latter is a counterpart of the SIN/G mutant harbouring a ^726^P→G mutation, allowing persistent non-cytopathic replication of SIN RNA replicons and genomes in BHK-21 cells (Frolov *et al.*, 1999; Gorchakov *et al.*, 2004).

To test the effect of these mutations on SFV viability, the corresponding replicons expressing the d1EGFP marker were transfected into BHK-21 cells. d1EGFP expression at 37 °C occurred only in cells transfected with replicons harbouring PG, RDR and RRD mutations. Replicons with ΔΔR, ΔRR, RDD and DDR mutations showed a temperature-sensitive phenotype; almost 100 % of transfected cells were d1EGFP-positive for all of these constructs at the permissive temperature (28 °C) (data not shown). In order to include these replicons and corresponding viruses in the analysis, subsequent assays were carried out at 28 °C. In contrast to mutants described above, at most a few cells expressed d1EGFP following transfection with SFV1-d1EGFP-DDD, and no d1EGFP expression was detected following transfection with SFV1-d1EGFP-ΔΔΔ (data not shown).

### Subcellular localization of nsP2 expressed by mutant SFV genomes

**Fig. 1. f1:**
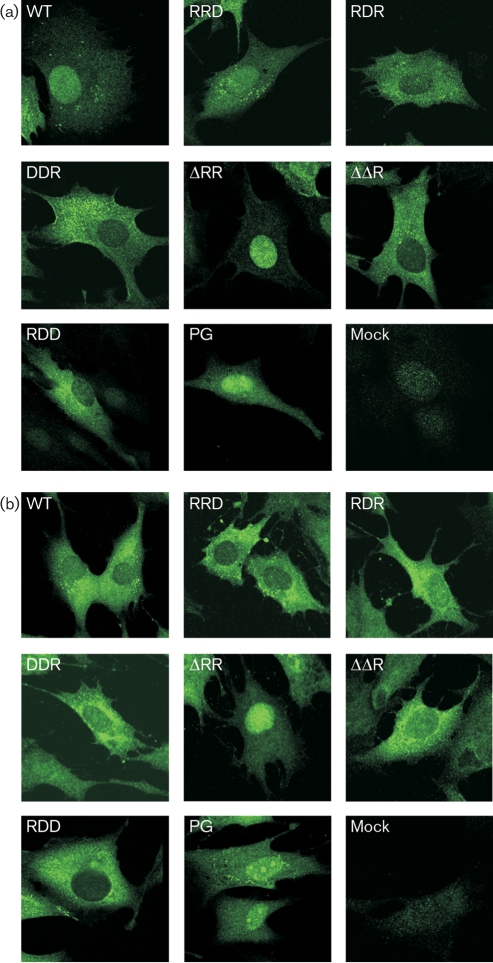
Subcellular localization of nsP2 in BHK-21 cells infected with SFV4 or its derivatives encoding the mutant nsP2 variants (corresponding mutations are indicated in each panel). The cells were fixed at 8 (a) or 24 (b) h p.i. and the localization of nsP2 was revealed by Alexa Fluor 488 anti-nsP2 staining.

Subcellular localization of nsP2 in cells infected with SFV4, SFV4-ΔΔR, SFV4-ΔRR, SFV4-DDR, SFV4-RDD, SFV4-PG, SFV4-RRD or SFV4-RDR was detected at 8 and 24 h p.i. nsP2 was found exclusively in the cytoplasm of SFV4-RDD-infected cells both 8 and 24 h p.i. (Fig. 1a, b[Fig f1]). Localization of nsP2 in SFV4-DDR- and SFV4-ΔΔR-infected cells was exclusively cytoplasmic at 8 h p.i. (Fig. 1a[Fig f1]) and mostly cytoplasmic at 24 h p.i. (Fig. 1b[Fig f1]), whereas in SFV4-RDR-infected cells there was some nuclear localization of nsP2 at both times p.i. In SFV4-ΔRR- and SFV4-PG-infected cells, nsP2 was almost entirely nuclear (Fig. 1a, b[Fig f1]). The localization of nsP2 in cells infected with SFV4-RRD resembled SFV4-infected cells and changed from mainly nuclear during early infection to predominantly cytoplasmic later (Fig. 1a, b[Fig f1]).

### Processing of non-structural polyproteins by mutant viruses

**Fig. 2. f2:**
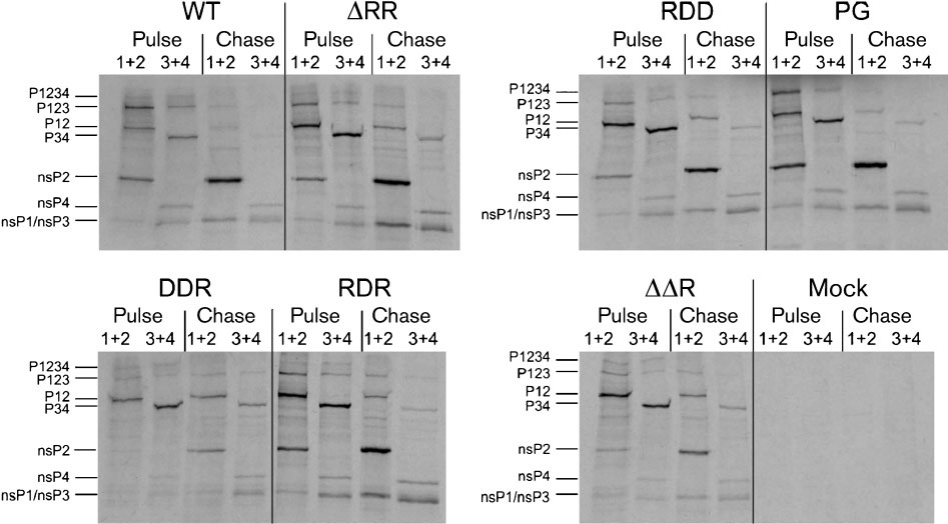
Analysis of the processing of non-structural polyproteins of the recombinant viruses. Pulse labelling was performed with [^35^S]methionine for 0.5 h; half of the samples were then chased with an excess of cold methionine for 1 h. The mutation introduced into the corresponding virus genome and the antibody combination used for immunoprecipitation (1+2 and 3+4 designate antibodies against nsP1 and nsP2 or nsP3 and nsP4, respectively) are indicated above each panel. The positions of proteins and polyprotein precursors are indicated on the left.

The lack of cleavage between nsP2 and nsP3 prevents translocation of nsP2 to the nucleus (Gorchakov *et al.*, 2005; Salonen *et al.*, 2003), whereas introduction of the RDR mutation into nsP2 results in slower non-structural-polyprotein processing (Fazakerley *et al.*, 2002; Rikkonen, 1996). To determine the effect of the NLS mutations on non-structural polyprotein processing, pulse–chase labelling followed by immunoprecipitation was carried out. The only difference compared with SFV4 was a small delay in P12 and P34 processing observed for SFV4-DDR and SFV4-ΔΔR at 8 h p.i. (Fig. 2). Thus, the detected slow-down of processing may have contributed to the change of nsP2 localization observed for these viruses from strictly cytoplasmic to partially nuclear (Fig. 1a, b[Fig f1]).

### Effect of mutations in the NLS of nsP2 on cytopathogenicity of SFV infection

The partial transport of nsP2 into the nucleus of SFV-infected cells has been proposed as one possible mechanism determining the cytopathogenicity of SFV infection (Peranen *et al.*, 1990; Rikkonen, 1996). Based on this assumption, NLS mutations that prevent nsP2 movement to the nucleus of infected cells should also reduce SFV cytopathogenicity.

**Fig. 3. f3:**
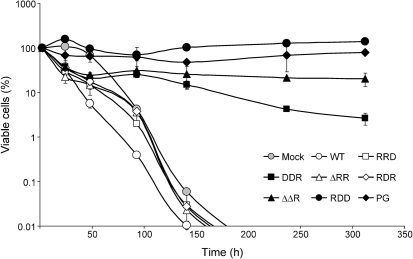
Survival of BHK-21 cells transfected with SFV1-Pac replicon or its derivatives with mutations in nsP2. Puromycin selection was applied starting from 16 h p.t. The percentages of viable adherent cells at 24, 48, 93, 141, 237 and 312 h p.t. were normalized by viable cell numbers detected at 2 h p.t. Results are shown as means±sd of two experiments.

SFV1-Pac-type replicons were used to analyse the cytotoxic effect of SFV replicons with mutations in nsP2. Puromycin selection was applied from 16 h p.t. to ensure that transfected cells had established a puromycin-resistant phenotype. The percentage of viable adherent cells was determined at 24, 48, 93, 141, 237 and 312 h p.t. (Fig. 3). Based on the data obtained, the mutant SFV replicons were divided into two groups. The first group was SFV1-Pac, SFV1-Pac-RRD, SFV1-Pac-RDR and SFV-Pac-ΔRR, with highly cytotoxic replication. SFV1-Pac-RDD, SFV1-Pac-PG, SFV1-Pac-DDR and SFV1-Pac-ΔΔR formed the second group and had significantly diminished cytotoxicity. The number of viable cells in this group was stable or slightly increased (SFV1-Pac-RDD and SFV1-Pac-PG), or decreased slowly (SFV1-Pac-DDR and SFV1-Pac-ΔΔR) (Fig. 3).

**Fig. 4. f4:**
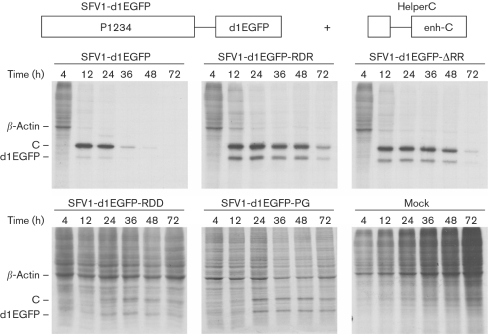
Effects of SFV1-d1EGFP, SFV1-d1EGFP-RDR, SFV1-d1EGFP-ΔRR, SFV1-d1EGFP-RDD and SFV1-d1EGFP-PG replicons on host-cell protein synthesis. BHK-21 cells were co-electroporated with transcripts from pSFV1-d1EGFP-based plasmids (50 μg) and from pHelperC (50 μg) and labelled metabolically at 4, 12, 24, 36, 48 and 72 h p.t. Equal amounts of cell lysate were analysed by SDS-PAGE, followed by autoradiography. The positions of d1EGFP, *β*-actin and the capsid protein (C) are indicated.

Changes in metabolism leading to the death of SFV-infected cells include virus-induced inhibition of host-cell translation (Glasgow *et al.*, 1997). Metabolic labelling was used to study the effects of recombinant SFV replicons on host-cell-specific protein synthesis. BHK-21 cells were co-transfected with *in vitro*-synthesized RNAs of replicons expressing d1EGFP and HelperC RNA, and pulse-labelled at 4, 12, 24, 36, 48 and 72 h p.t. HelperC was used to gain information about translational efficiency from the subgenomic promoter located *in trans*. As expected, protein synthesis in cells transfected with SFV1-d1EGFP was rapidly inhibited in contrast to mock-infected cells (Fig. 4). Only slightly slower shutdown of host protein synthesis was detected in cells transfected with SFV1-d1EGFP-ΔRR, SFV1-d1EGFP-RDR (Fig. 4), SFV1-d1EGFP-DDR and SFV1-d1EGFP-ΔΔR transcripts (data not shown). In contrast, no decrease in host-cell translation was detected in SFV1-d1EGFP-RDD- and SFV1-d1EGFP-PG-transfected cells (Fig. 4).

**Fig. 5. f5:**
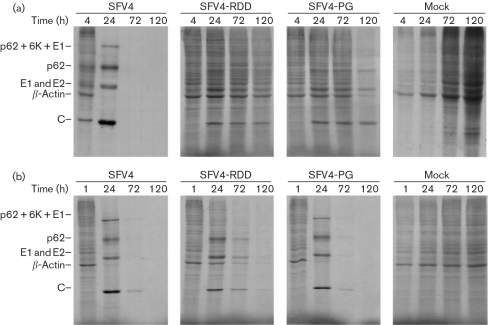
Effects of SFV4, SFV4-RDD and SFV4-PG on protein synthesis in BHK-21 cells following transfection with 50 μg *in vitro*-transcribed RNA (a) or infection with collected virions at an m.o.i. of 1 (b). The cells were labelled metabolically at 4, 24, 72 and 120 h p.t. or at 1, 24, 72 and 120 h p.i. Equal amounts of cell lysate were analysed by SDS-PAGE, followed by autoradiography. The positions of SFV structural proteins and cellular *β*-actin are indicated.

Structural proteins of alphaviruses participate in virus-induced cytotoxicity (Nieva *et al.*, 2004; Singh & Helenius, 1992; Wengler *et al.*, 2003). Therefore, the shutdown of host-cell translation was also analysed for cells transfected with transcripts from infectious cDNA clones containing mutations in the nsP2 region. The results of this experiment were very similar to those for the corresponding replicon vectors. Transfection of cells with SFV4 RNA caused rapid shutdown of host-cell protein synthesis, whilst transfection with SFV4-RDD or SFV4-PG RNA did not (Fig. 5a[Fig f5]). Thus, the presence and expression of structural genes did not induce shutdown of host-cell translation. In contrast, in cells infected with SFV4-RDD or SFV4-PG virions, shutdown of cellular translation was similar to that in SFV4-infected cells (Fig. 5b[Fig f5]). RT-PCR and sequencing were used to confirm the presence of the RDD and PG mutations, showing that the phenotypic change could not be attributed to reversion.

One possible explanation is that the shutdown of cellular translation depends on the pathway of mutant viral genome entry (transfection versus infection). Another possibility is that the cytotoxicity of mutant SFVs is restored by compensatory mutations. When BHK-21 cells were transfected with infectious RNAs purified from cells transfected with SFV4-RDD or SFV4-PG, shutdown of cellular translation occurred (data not shown). This indicated that alterations in RNA sequences, rather than the difference in mode of entry, resulted in the change in recombinant virus phenotype.

### Packing efficiency of mutant replicons correlates with the level of sgRNA-mediated protein expression

The ability of non-cytotoxic-alphavirus-based replicons to be packaged into VLPs by supplementing with helper RNAs for structural proteins can be significantly reduced (Perri *et al.*, 2000). In the present study, most replicons with mutations in the NLS of nsP2 were packaged into VLPs as efficiently as the parental SFV1-d1EGFP: typically approximately 250 VLPs were obtained per SFV1-d1EGFP-transfected cell, whilst the yield ranged from 200 to 270 VLPs per transfected cell for SFV1-d1EGFP-RRD, SFV1-d1EGFP-RDR, SFV1-d1EGFP-DDR, SFV1-d1EGFP-ΔRR and SFV1-d1EGFP-ΔΔR. The exceptions were SFV1-d1EGFP-RDD and SFV1-d1EGFP-PG, which generally produced less than one VLP per transfected cell. As neither mutation overlapped the SFV packaging signal (White *et al.*, 1998), we analysed mutant replicons for defects in structural protein expression and/or replicon RNA production.

Metabolic labelling (Fig. 4) showed different expression levels of *in trans*-encoded capsid protein and *in cis*-encoded d1EGFP, translated from sgRNAs. Capsid protein was expressed most efficiently (albeit briefly) in cells where HelperC RNA was co-transfected with SFV1-d1EGFP RNA. In the same cells, the expression of d1EGFP marker, which unlike capsid protein did not have the translational enhancer, was the lowest and the shortest (Fig. 4). In cells co-transfected with SFV1-d1EGFP-RDR or SFV1-d1EGFP-ΔRR, capsid protein expression lasted until 72 h p.t., and in cells co-transfected with SFV1-d1EGFP-RDD or SFV1-d1EGFP-PG, capsid protein continued to be expressed at low levels up to 72 h p.i. The expression profile of d1EGFP protein was very similar to that of capsid protein in all these cells (Fig. 4). Thus, the expression of marker proteins depended on the presence or absence of the capsid enhancer sequence and the ability of the replicon to shut down cellular translation. The *cis*/*trans* localization of the marker gene may have affected translational efficiency.

**Fig. 6. f6:**
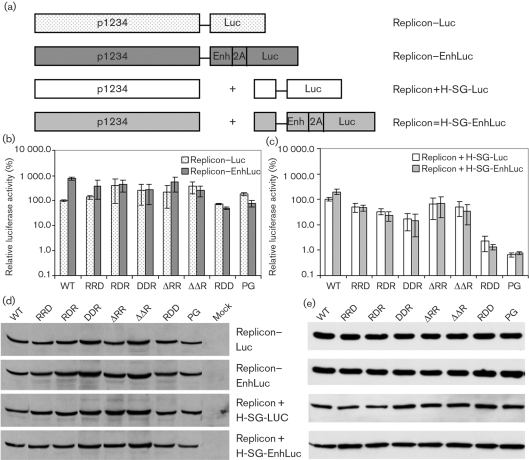
Expression of the luciferase marker by mutant replicons. (a) Schematic presentation of the *in cis* and *in trans* expression systems. (b) Luciferase activity measured in cells transfected with SFV replicon vectors expressing luciferase (activity *in cis*). Relative luciferase activities are normalized to that of SFV1-Luc (WT). Results are shown as means±sd of three experiments. (c) Luciferase activities measured in cells transfected with SFV replicon vectors and luciferase-encoding helper RNAs (activity *in trans*). Relative luciferase activities are normalized to that of SFV1 (WT)+H-SG-Luc. Results are shown as the means±sd of three experiments. (d) Western blot analysis using anti-nsP1 antibody of extracts from transfected cells. (e) Western blot analysis using anti-*β*-actin antibody of extracts from transfected cells.

Expression from sgRNAs was evaluated in detail using a reporter system with a luciferase marker expressed from a subgenomic promoter, located either on the replicon RNA (*in cis*) or on the separate helper RNA H-SG-Luc (*in trans*). The impact of the capsid enhancer sequence on the expression of marker proteins was also studied. Western blot analysis of transfected cells revealed that all mutant replicons produced roughly equal amounts of non-structural proteins (Fig. 6d[Fig f6]). Similarly, in the absence of translational enhancer, expression levels of the luciferase marker from *in cis* subgenomic promoters by mutant replicons were similar to or higher than those of SFV1-Luc (Fig. 6b[Fig f6]). However, from the *in trans* subgenomic promoter, the levels of luciferase activity with mutant replicon transfections were reduced compared with SFV1-MCS. The reduction was greatest for the SFV1-MCS-RDD and SFV1-MCS-PG replicons (Fig. 6c[Fig f6]). The addition of a translational enhancer increased luciferase expression approximately 10-fold in the case of transfection with wild-type replicons, in agreement with Sjoberg *et al.* (1994). In contrast, the enhancer had no effect for most mutant replicons and was negative for SFV1-EnhLuc-RDD and SFV1-EnhLuc-PG (Fig. 6b, c[Fig f6]). Thus, one reason for low efficiency of VLP formation of RDD and PG mutants could be significantly decreased protein expression from the subgenomic promoter on the Helper1 RNA.

### RDD and PG mutations severely reduce the levels of SFV RNA accumulation

Several studies have shown that substitutions in nsP2 resulting in decreased cytopathogenicity or persistent infections of corresponding alphavirus-based replicons also downregulate replicon RNA replication (Agapov *et al.*, 1998; Dryga *et al.*, 1997; Frolov *et al.*, 1999; Perri *et al.*, 2000; Sawicki *et al.*, 2006). In the present study, we determined levels of positive-strand (genomic and subgenomic) RNA synthesis in BHK-21 cells co-transfected with either SFV1-MCS-RDD and H-SG-EnhLuc or SFV1-MCS-PG and H-SG-EnhLuc transcripts using Northern blotting. Transcripts of SFV1-MCS, SFV1-MCS-RRD and SFV1-MCS-ΔRR were included as controls.

**Fig. 7. f7:**
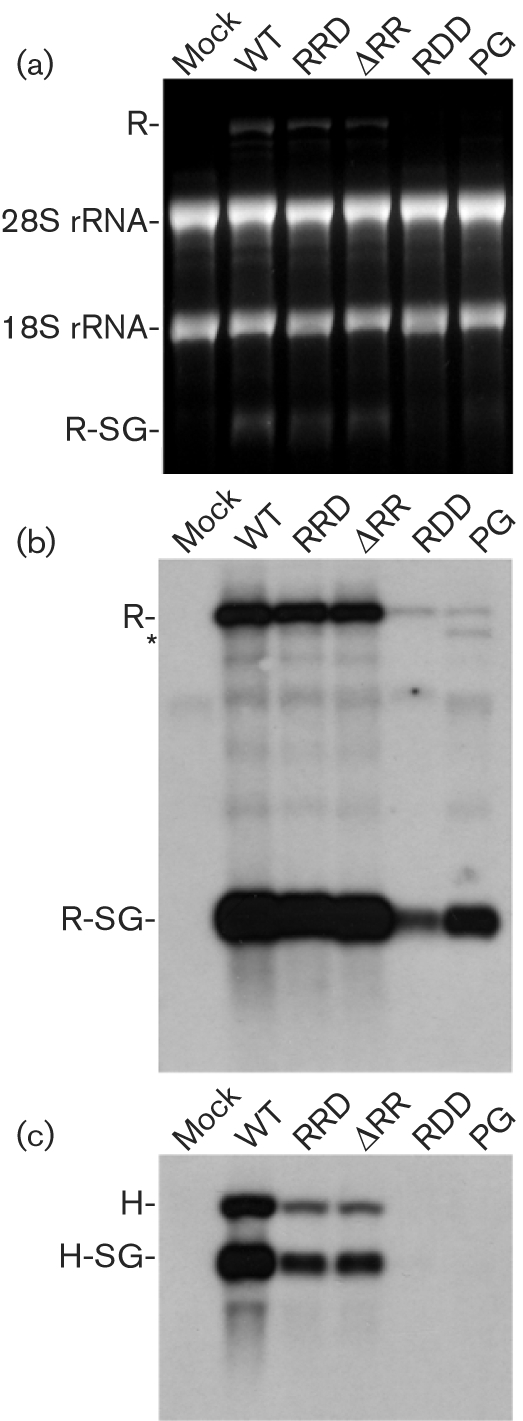
Accumulation of positive strands of replicon and helper RNAs and corresponding sgRNAs in BHK-21 cells co-transfected with SFV1-MCS replicon RNAs and H-SG-EnhLuc helper RNA at 24 h p.t. (a) Fragment of gel stained with ethidium bromide. (b) Northern blotting with RNA probe complementary to the 3′ end of the replicon RNA and the corresponding sgRNA. The positions of corresponding RNA molecules are indicated. R indicates the replicon RNA and R-SG indicates corresponding sgRNA. An asterisk indicates defective interfering RNA detected in cells transfected with SFV-MCS-PG. (c) Northern blotting with RNA probe complementary to the luciferase-encoding region. Positions of H-SG-EnhLuc helper RNA and the corresponding sgRNA molecules are indicated.

The replication of replicon RNAs and transcription from the subgenomic promoter were strongly impaired for SFV1-MCS-RDD and SFV1-MCS-PG (Fig. 7b[Fig f7]). For both SFV1-MCS-PG and SFV1-MCS-RDD, the amount of replicon RNA was at least 30-fold lower than for SFV1-MCS. The amount of sgRNA synthesized by SFV1-MCS-RDD and SFV1-MCS-PG was reduced to a lesser extent as there was a 3–6-fold reduction at different times. The molar ratio of sgRNAs and genomic RNA was also changed (Fig. 7b[Fig f7]), from typically 15 : 1 for SFV1-MCS, SFV1-MCS-RRD and SFV1-MCS-ΔRR to at least 100 : 1 for SFV1-MCS-RDD and SFV1-MCS-PG. Thus, the RDD and PG mutations in nsP2 affected genomic RNA synthesis more severely than that of sgRNA.

The synthesis of genomic RNAs and sgRNAs from promoters located on helper RNAs (*in trans*) was impaired more strongly by all mutations analysed in nsP2 (Fig. 7c[Fig f7]). Compared with SFV1-MCS, there was an approximately 4–6-fold reduction in synthesis of both genomic and sgRNA for H-SG-EnhLuc co-transfected with SFV1-MCS-RRD or SFV1-MCS-ΔRR. RDD and PG substitutions in nsP2 severely inhibited the synthesis of genomic and sgRNAs from H-SG-EnhLuc; the corresponding products could not be detected (Fig. 7c[Fig f7]). Thus, sgRNA expression correlated well with marker protein expression. In the case of SFV1-Luc-PG and SFV1-Luc-RDD, the reduction in sgRNA synthesis is compensated by the lack of translational shutdown. In contrast, the structural protein expression from the helper RNAs is severely downregulated due to suppression of synthesis of the corresponding sgRNAs and the lack of translational shutdown needed to activate the capsid enhancer element.

## DISCUSSION

SFV nsP2 is the key regulator of the viral replication cycle and is important for virus–host interactions. For the Old World alphaviruses, nsP2 is also crucial for virus-induced cytotoxicity (Garmashova *et al.*, 2007), shutting down cellular transcription and translation and inducing cell death. The mechanisms and pathways of these effects are poorly understood, but events in the nucleus of the infected cell may be important. The partial transport of nsP2 into the nucleus of SFV-infected cells could trigger the mechanisms determining the cytopathogenicity of SFV infection (Peranen *et al.*, 1990; Rikkonen, 1996). In the present study, we analysed the effect of mutations in the C-terminal domain of nsP2, mostly in the NLS sequence, on the cytotoxicity of SFV genomes and replicons.

The present study indicated that the NLS of nsP2 is crucial and multifunctional. Changes of single amino acid residues in the region were tolerated, but deletions of one or two arginine residues or changes of two arginine residues always resulted in a temperature-sensitive phenotype. Three changes drastically reduced infectivity, but the mutant replicon was still able to replicate in a few transfected cells. Deletion of all three arginines was lethal and no replication occurred. The nature of these defects was not analysed in detail, but were most likely defects in the viral replicase complex formation and/or functioning. As temperature-sensitive mutations in nsP2 of SFV have defects in protease function (Balistreri *et al.*, 2007), it is possible that the protease activity of nsP2 may be one function affected by NLS mutations.

Similar to previous studies (Rikkonen, 1996; Rikkonen *et al.*, 1992, 1994b), it was found that the RRD mutation in the NLS did not prevent nsP2 transport to the nucleus, whereas localization of nsP2 with an RDR, DDR, ΔΔR or RDD mutation was mostly or exclusively cytoplasmic. However, deletion of one arginine residue in the NLS resulted in enhanced nuclear transport of the nsP2, similar to SFV4-PG-infected cells. Previous studies (Rikkonen, 1996) have shown that nsP2 expressed by SFV4-RDR is exclusively cytoplasmic. In contrast, some nuclear localization was detected in this study (Fig. 1a, b[Fig f1]). It is possible that a delay in non-structural polyprotein processing, observed for SFV4-RDR at 37 °C (Rikkonen, 1996) but not at 28 °C (Fig. 2), contributed to that difference.

There was no strict correlation between nuclear localization of nsP2 and cytotoxicity. The nsP2 proteins encoded by mutant replicons with cytotoxicity retained some nuclear localization (Figs 1 and 3); however, one of the least cytotoxic mutants, SFV4-PG (Figs 3–5), had enhanced transport of nsP2 into the nucleus (Fig. 1). The differences in cytotoxicity of SFV replicons cannot be explained by the levels of nsP2 expression, as all replicons expressed non-structural proteins at very similar levels (Fig. 6d[Fig f6], data not shown for nsP2–4).

Synthesis of viral RNA is one factor that may contribute to virus-induced cytotoxicity. Accordingly, synthesis of positive-strand RNAs in cells co-transfected with different replicons and H-SG-EnhLuc RNA was analysed. For SFV1-MCS-RDD and SFV-MCS-PG, the accumulation of replicon RNA was severely reduced (Fig. 7b[Fig f7]), indicating that the RDD and PG mutations affected transcription from the genomic promoter. A similar reduction in genomic RNA synthesis has been reported for persistently replicating SFV and SIN replicons with different mutations in nsP2 (Sawicki *et al.*, 2006). In the present study, sgRNA synthesis was less affected and thus the molar ratio of genomic : sgRNA was drastically changed. Again, these data are consistent with previous results on SIN and SFV replicons with mutations in the nsP2 region (Frolov *et al.*, 1999; Perri *et al.*, 2000).

Packaging of alphavirus-based replicon vectors relies on the ability of the viral replicase to replicate and transcribe separately provided helper RNA(s). In the present study, all mutant SFV replicons had reduced ability to replicate templates provided *in trans*. For SFV1-MCS-RRD and SFV1-MCS-ΔRR replicons without obvious defects in self-replication, the ability to replicate and transcribe H-SG-EnhLuc RNA was reduced 4–6-fold; *in trans* activity of the replicases encoded by SFV1-MCS-PG and SFV1-MCS-RDD was below the detection limit (Fig. 7c[Fig f7]). This finding, combined with the severely reduced replication of their replicon RNAs, may explain why SFV1-MCS-PG and SFV1-MCS-RDD were extremely inefficient in VLP production.

In part, the properties of SFV1-RDD and SFV1-PG could be due to the fact that non-cytotoxic replicons of SIN and SFV are unable to make stable replicase complexes and exhibit continuous negative-strand RNA synthesis (Sawicki *et al.*, 2006). Consequently, the synthesis of non-structural polyprotein and, importantly, its processing into the functional early replicase complex (P123+nsP4) should also be continuous. As a result, in cells infected with replicons containing RDD or PG mutations, the high levels of free nsP2 should greatly reduce the formation of functional replicase complexes by cleaving the non-structural polyprotein between nsP2 and nsP3. This should become especially crucial for formation of functional replicase complexes on helper RNA, as relocalization of the unprocessed non-structural polyprotein would be needed. The findings that replication levels of SFV1-RDD and SFV1-PG were low and that these constructs were severely defective in replication of co-transfected helper RNAs are coherent with this hypothesis.

Another property of the SFV replicons and genomes with PG and RDD mutations was apparent inhibition of transfected cell division. This was evident from the lack of increase of host protein expression in cell cultures transfected with SFV1-d1EGFP-PG, SFV1-d1EGFP-RDD, SFV4-PG or SFV4-RDD (Figs 4 and 5). Additionally, the number of cells transfected with SFV1-Pac-RDD and SFV1-Pac-PG remained almost unchanged, even after prolonged incubation (Fig. 3), and cells transfected with SFV1-Pac-RDD and SFV1-Pac-PG formed very few colonies under puromycin selection at 28 °C (data not shown). In contrast, highly efficient colony formation has been reported for cells transfected with non-cytotoxic SIN replicons (Frolov *et al.*, 1999; unpublished data).

Both SFV4-PG and SFV4-RDD viruses converted the phenotype from non-cytotoxic (Fig. 5a[Fig f5]) to cytotoxic (Fig. 5b[Fig f5]). This differed from SIN/G virus, which maintains non-cytotoxic properties (Gorchakov *et al.*, 2005). The conversion was accompanied by increased synthesis of viral structural proteins (Fig. 5b[Fig f5]) and was not caused by reversion of the original mutations. The conversion was possibly due to the advent, selection and accumulation of compensatory mutations. The accumulation of SFV genomes with compensatory mutations has been found in response to low initial infectivity of mutant genomes (Zusinaite *et al.*, 2007). However, in the present study, SFV4-PG and SFV4-RDD did not have defects in infectivity; both *in vitro*-transcribed replicon RNAs and full-length genomic RNAs resulted in >95 % transfected cells (data not shown). Therefore, low efficiency of virion formation is the most likely defect causing accumulation of compensatory mutations. This assumption can also explain why the properties of SIN/G differ from those of SFV4-RDD and SFV4-PG. The level of RNA replication and structural protein expression of SIN/G is relatively high (Gorchakov *et al.*, 2005) and there was no selection pressure for genotypes that formed more particles than the original mutant.
